# Power transformation for enhancing responsiveness of quality of life questionnaire

**DOI:** 10.1186/s40064-015-1296-9

**Published:** 2015-09-25

**Authors:** YanYan Ange Zhou

**Affiliations:** Yale School of Management, New Haven, CT 06510 USA; California State University, East Bay, Hayward, CA 94542 USA

**Keywords:** Box–Cox transformation, Paired t-test, Responsiveness, Asthma quality of lfe questionnaire (AQLQ)

## Abstract

We investigate the effect of power transformation of raw scores on the responsiveness of quality of life survey. The procedure maximizes the paired t-test value on the power transformed data to obtain an optimal power range. The parallel between the Box–Cox transformation is also investigated for the quality of life data.

## Background

Symptom and quality of life (QOL) instruments are often used in clinical trials to reach significant conclusions. However, these instruments need to be validated (with respect to their measurement properties as construct validity, reliability, reproducibility, discriminant ability and/or responsiveness) before they can be used to judge statistical significance. Ordinal scores are often assigned. For example, the Quality of Life Instrument breast cancer patient version is a 46 item ordinal scale ranging from 0 = worst outcome to 10 = best outcome. Asthma quality of life questionnaire (AQLQ) is often scaled from 1 to 7 with higher score reflects better functions. When baseline scores are available, common sense dictates that baseline values should be incorporated in the analysis in order to improve efficiency and reduce imbalances. However, it is not immediately clear how the baseline values should be used in the statistical analysis. One of the popular ways of analyzing the scores from baseline and outcome is to use nonparametric methods (such as Wilcoxon signed-rank test or Wilcoxon rank-sum test) which uses the rank of the raw scores. Since no assumptions about the probability distributions are made, such nonparametric methods are meaningful but could have considerably less power. The other popular avenue is to analyze the raw scores as change or percent change from the baseline. Some references in the medical literature, by no means complete, include Pearlman et al. (1992), Juniper et al. (1993), Chervinsky et al. (1994), Israel et al. (1996). There are analysis using number of days without symptoms (where daily recordings are used for a binary classification: 1 if no/nominal symptom, 0 otherwise) as the observations; see Pearlman et al. (1992), Dahl et al. (1994). There is a body of literature which points out the advantages of using baseline scores as covariates; see Blomqvist (1977), Salsburg (1981), Hayes (1988), Kaiser (1989), and Senn (1989) and the references therein. Symptom or QOL scores are often analyzed using linear models (e.g., ANOVA or Cochran–Mantel–Haenszel test using the assigned scores); see Agresti (1990), Canover (1980). Stratified analysis based on baseline strata can give information about how the responses differ for different baseline values and whether pooling or ignoring baseline values makes sense. It is evident from the above discussion that there are many methods for analyzing QOL data and no standards are implemented in practice. A major drawback of methods that use the raw scores rather than the ranks is that they can depend critically on the scale used for the score assignment. For example, a score assignment from 0 to 4 may have a difference of 2 between the scores of moderate to maximum symptom whereas it is a difference of 3 for a score assignment of 0–6. Thus, the significance results will be highly sensitive to the particular scale used for score assignment. The results can mislead the reader about the treatment magnitudes and make it nearly impossible to compare across studies. The analysis can induce outliers.

*Responsiveness*—the sensitivity of a measure to a clinically relevant change in health is an essential property of outcome measures for intervention studies. The main objective of this paper is to study the responsiveness to change of the assignment of the scores in the AQLQ measure. We do this under the paradigm of power group of transformation, by studying the sensitivity of the methods for validating QOL instruments to the assignment of the scores.

There is no consensus regarding how best to assess the responsiveness to change of measures; here we looked at responsiveness as measures of treatment effect. Such measures tell us little about how well the instrument serves its purpose, which is not our objective; but are of customary use in interpreting score changes (Terwee et al. 2003). We look at several existing tests and also suggests some new tests and study how the results vary as we change the scale of measurements. We want to reiterate that it is not our objective to build new methodologies that circumvent the problem of assignment of scores (even though we do define alternative methods, but only to bring more clarity to our investigation) but rather point out the deficiencies that plague some of the commonly used methods and how sensitive they are to the actual scale of measurements. In “Within treatment comparison”, we describe the data and the treatments. We also investigate the susceptibility of statistical conclusions about within treatment comparisons when different methods are used for power transformation. We look at the problem from two different objectives, one is to transform the data in order to achieve normality (which is the underlying assumption of the quantitative analysis) and the other to get the most significant test statistic. In “Between treatment comparison”, we perform similar analysis regarding the sensitivity of the between treatment comparisons to the power transformation. We also use the novel method of generalized confidence interval to make the sensitivity analysis under unequal treatment variances. In “Conclusion”, we summarize our results and provide some recommendation.

## Within treatment comparison

In this section we use a dataset from the ‘Quality of Life’ survey of an undisclosed clinical study to perform our investigation and to illustrate our methodology.

### Quality of life data

There were a total of 689 asthma patients undergoing the trial. Subjects were non-smokers aged 15–70 years with ≥1 year history of asthma symptoms who met the inclusion criteria. Patients were excluded if they have other pulmonary disorder, emergency treatment for asthma within 1 month, hospitalization within 2 months or respiratory tract infection within 3 weeks. The eligible patients were randomized to each of the four treatments: 2A = M/UC, 2B = M/M, 2C = P/UC and 2D = P/M where M, P and UC stand for the active drug (Montelukast), placebo and usual care, respectively and A/B stands for application of treatment A followed by treatment B.

For each patient the baseline responses and post treatment responses for the AQLQ with 13 items were recorded. The subsequent outcomes after the baseline observations were recorded in a series of visits over the entire period of the study. For illustrative purposes we have chosen outcome values only from the first visit (visit = 6) after the baseline observation (visit = 3). All subjects for whom complete data records were available were included in this analysis (632 subjects out of 689). There were 57 subjects excluded due to missing the first post dose visit. Among the subjects who had both visits, the treatment group sizes were $n_1 = 146,\,\, n_2 = 274,\,\, n_3 = 70\,\,{\text{and}}\,\,n_4 = 142.$

The asthma specific QOL questionnaire can be further classified into two domains, one corresponding to activity level and the other corresponding to emotional level. For preliminary analysis we have ignored this further grouping. Thus the observations are $x_{ijkl}, \; i=1,\ldots ,4, \; j=1,\ldots ,n_i,\; k = 1,\ldots ,13, \; l=0,1,$ corresponding to the answer to the *k*th question in the *l*th period (l = 0 for baseline and l = 1 for post treatment) for the *j*th patient in the group receiving the *i*th treatment.

### Paired comparison

The baseline and outcome values within a patient are correlated and can be thought of as matched groups. A suitable test for dependent categorical variables can be used for finding out whether there is any difference in the baseline and the outcome distribution within treatments. One can also treat the observations as continuous values and perform a paired t-test to test for differences between pre and post treatment responses. Of course, when the data are truly continuous and normal, the paired t-test has optimality property such as most powerful unbiased test. Thus, one recourse could be to first try to transform the data to normal by means of transformations, such as the power group of transformation proposed by Box–Cox.

### Wilcoxon signed rank test

The Wilcoxon signed rank test can be used to test for symmetry around zeros of the difference between the outcome and baseline within a treatment group; see Agresti (1990). In Table 1 we tabulate the normalized test statistic value of the Wilcoxon signed-rank test and the corresponding p value obtained from the asymptotic null distribution for each treatment group. The observations used the difference between the outcome and the baseline of the average of the 13 questionnaire scores for each patient within a treatment group.

**Table 1:** Wilcoxon signed-rank test

Treatment group	*W*	p value
2A	5.77	<0.00001
2B	5.94	<0.00001
2C	2.40	0.01630
2D	3.51	0.00040

Even though all treatment groups give significant results, clearly the two groups associated with placebo are less significant than the two groups associated with the treatment.

### Box–Cox transformation

In Box–Cox transformation the transformed variables are

**Fig. 1:**
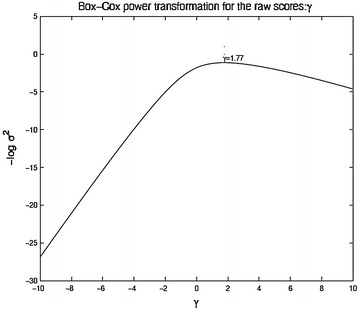
Box–Cox trans

2.1 $$\begin{aligned} y_i = {\left\{ \begin{array}{ll} \frac{\log x_i}{g^{\lambda - 1}} &  \lambda = 0 \\ \frac{x_i^{\lambda } - 1}{\lambda g^{\lambda - 1}} &  \lambda \ne 0, \end{array}\right. } \end{aligned}$$

where *g* is the geometric mean of the observations $g = (\prod x_{i})^{1/n}$. The data dependent constant $g^{\lambda - 1}$ comes in as the Jacobian of the power transformation. Then one looks at the variance of the transformed observations to choose an optimal value for $\lambda$. Figure 1 plot the negative of the log variance of the transformed observations as a function of the power $\lambda$ in a region $\lambda \in [-6,6]$. The value of $\lambda$ that minimizes the variance is approximately ${\lambda }_{max} = 1.77$ for both treatments 2B and 2C. Now if the paired *t*-test is performed with the transformed data, the absolute values of the *t* statistic are 4.7 and 14.5 for treatments 2B and 2C, respectively.

### Most significant paired *t*-statistic

**Fig. 2:**
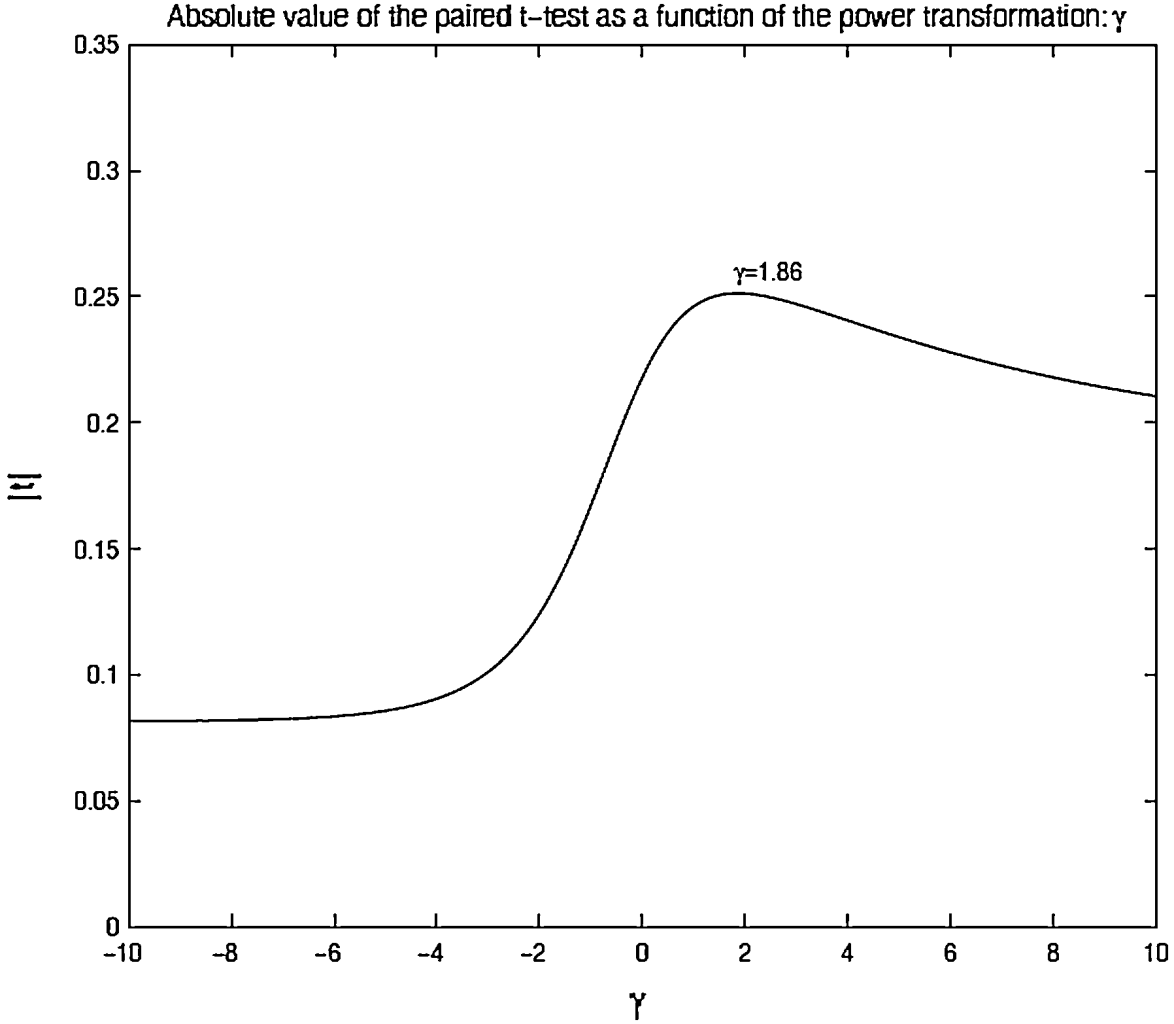
Paired *t*-statistic

Another way of approaching the problem of testing is maximizing the paired t-test value (in absolute terms) over different power transformations. The rational is to transform the scoring system to obtain most efficiency in detecting mean differences. This will change the type I error rate along with the power of the test. The observed mean differences for each patient receiving treatment *i* are $d_{ij} = 13^{-1}\sum _{k=1}^{13}(x_{ijk1}-x_{ijk0}), \;\; j = 1,2,\ldots ,n_i$. The paired t-test for the group receiving treatment *i* is then $t = {\sqrt{n_i}}{\bar{d}}/s_d$ where ${\bar{d}}$ is the mean of the observed $d_{ij}$ and $s_d$ is the sample standard deviation of the $d_{ij}$. We propose to transform the raw scores $x_i$ to $y_i$ by the transformation (2.1) and compute a paired t-test value $t(\lambda )$ for each value of $\lambda$ and choose our estimator for the exponent as ${\lambda }_{max} = argmax |t(\lambda )|$. Note that this simply entails transforming the data to $x_{ijkl}^{\lambda }$ as the *t* function is invariant to transformation of the form $a x_{ijkl} + b$. In Fig. 2 we present the absolute value of the paired *t* statistic for the treatment groups receiving treatment 2B and 2C. For treatment 2B the maximum of the *t* value is obtained at $\lambda = 1.86$ which is very close to that obtained through the Box–Cox transformation, but not exactly the same. A justification of this approach can be that because the exact type I error is not known, the objective of maximizing power can be directly obtained through the class of transformation that is often used to get more normal looking data, a case desirable for optimality of the paired t-test. The exponent that maximizes the power for treatment group corresponding to 2C is 3.5 which is substantially different from the value required for achieving normality. However, the *t* values with or without transformation are all significant for both groups. The group (2C) getting the placebo still shows significant difference from the baseline to the outcome. An explanation may be that the mere fact that a person is undergoing a trial has a psychological effect which generates this difference.

The results from the paired *t*-tests after transformation shows the difference in the grading from baseline to outcome for different treatments more markedly than the Wilcoxon signed-rank tests.

## Between treatment comparison

In this section we do pairwise comparison of the treatment effects. First we provide results for nonparametric tests for treatment differences. Then we investigate the effect of power transformation on parametric procedures for testing treatment differences.

### Wilcoxon rank-sum test

The data vectors for each treatment groups are the $n_i$ average score differences between the baseline and outcome for that group. Because the observation lengths are different for different treatment groups, we do Wilcoxon rank-sum test for testing treatment differences. The results for the normalized Wilcoxon rank-sum statistics $[W - E(W)]/\sqrt{V(W)}$, where for comparing treatment A and B the expectation and the variance of the statistic are $n_A (n_A + n_B)/2$ and $n_A n_B (n_A + n_B)/12$, respectively, and the corresponding p value obtained from the asymptotic null distribution of the test statistic are given in Table 2.

**Table 2:** Wilcoxon rank-sum test *W*

Treatment pair	*W*	p value
2A, 2B	1.35	0.175
2A, 2C	−1.95	0.051
2A, 2D	−1.84	0.066
2B, 2C	−0.93	0.353
2B, 2D	−0.74	0.460
2C, 2D	−0.27	0.786

### Two sample *t*-test

For initial investigation of the effect of the power transformation on between treatment comparison, we chose to do pairwise analysis rather than multiple comparison. The test statistic for pairwise comparison of treatments *i* and *j* is a *t*-statistic with $n_i + n_j - 2$ degrees of freedom. We report the corresponding $F_{n_i + n_j -2}$ statistic obtained from the analysis of variance.

To correct the effect of the baseline, we looked at the difference of average score over the 13 questionnaires from baseline to post-treatment response. There are several issues one needs to consider before proceeding with treatment comparison based on the transformed data. Of course the baseline and post-treatment scores and different treatment scores may need different power transformation for optimal result. However, for different power transformation the scales will be different making treatment comparison infeasible.

To analyze the data in the original scale the observations are divided by the Jacobian of the transformation after the transformation (similar to what one would do for Box–Cox transformation). Thus the new transformed scores for comparing TrtA and TrtB are

3.2 $$\begin{aligned} {\tilde{x}}_{ijkl} = {\left\{ \begin{array}{ll} \frac{\log x_{ijkl}}{g^{\lambda - 1}} &{} \lambda = 0 \\ \frac{x_{ijkl}^{\lambda } - 1}{\lambda g^{\lambda - 1}} &{} \lambda \ne 0, \end{array}\right. } \end{aligned}$$

where *g* is the geometric mean of the observations $g = (\prod _{i=A}^{B}\prod _{j=1}^{n_i}\prod _{k=1}^{13}\prod _{l=0}^{1} x_{ijkl})^{1/n}$ and $n = n_A + n_B, \;(A,B) \in \{ 1,2,3,4\}$. Final analysis is done on the reduced data (averaged over the questionnaires) and taking difference from the baseline to the post-treatment values).

3.3 $$\begin{aligned} y_{ij} = 13^{-1} \sum _{k=1}^{13} x_{ijk1} - 13^{-1} \sum _{k=1}^{13} x_{ijk0}. \end{aligned}$$

As stated before, treatment comparison can be made based on a simple one-way model for $y_{ij}$,

3.4 $$\begin{aligned} y_{ij} = \mu + \tau _i + e_{ij}, \; i = A,B, \; j = 1,\ldots ,n_{i}. \end{aligned}$$

**Fig. 3:**
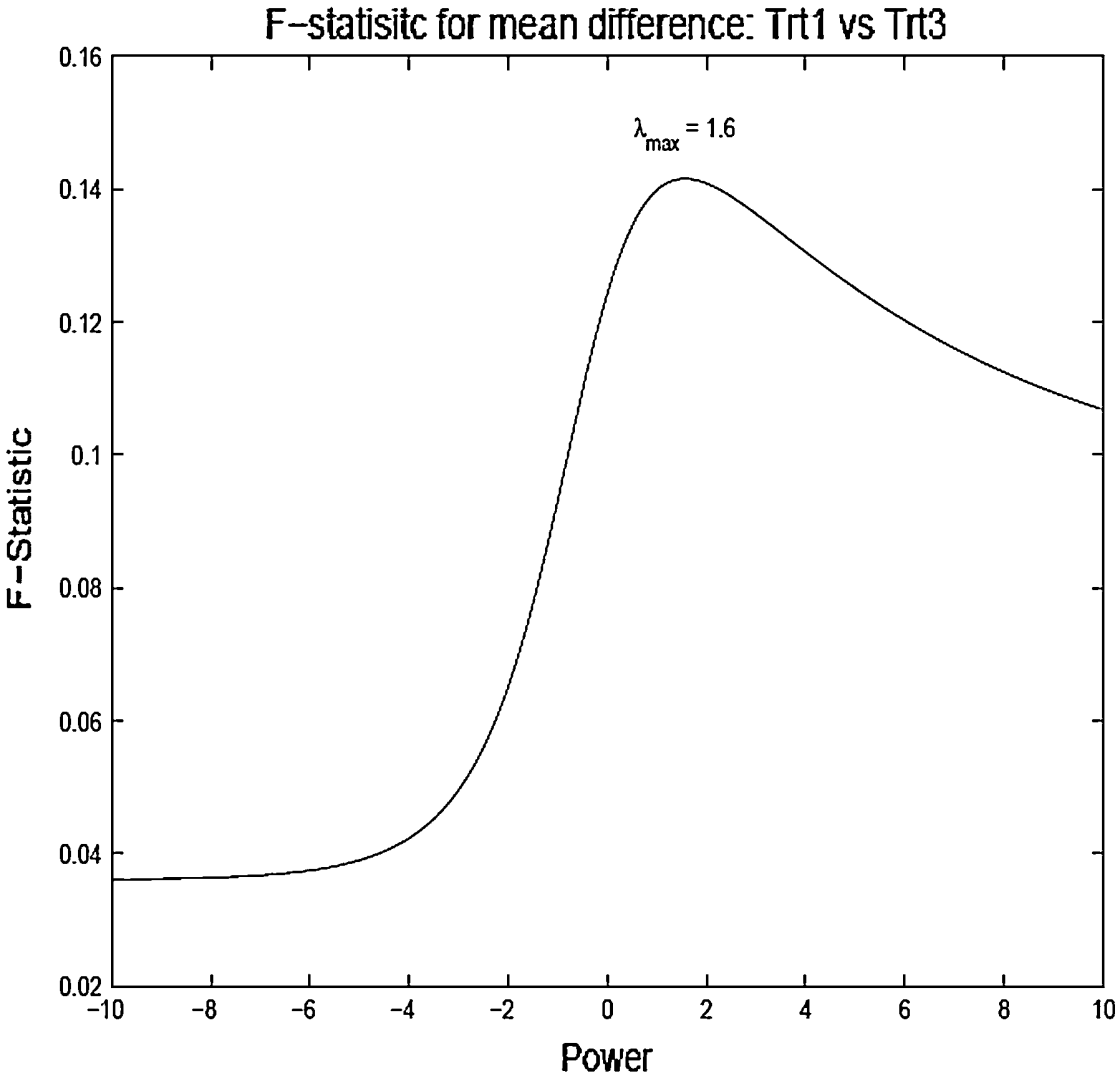
2A vs 2C

**Fig. 4:**
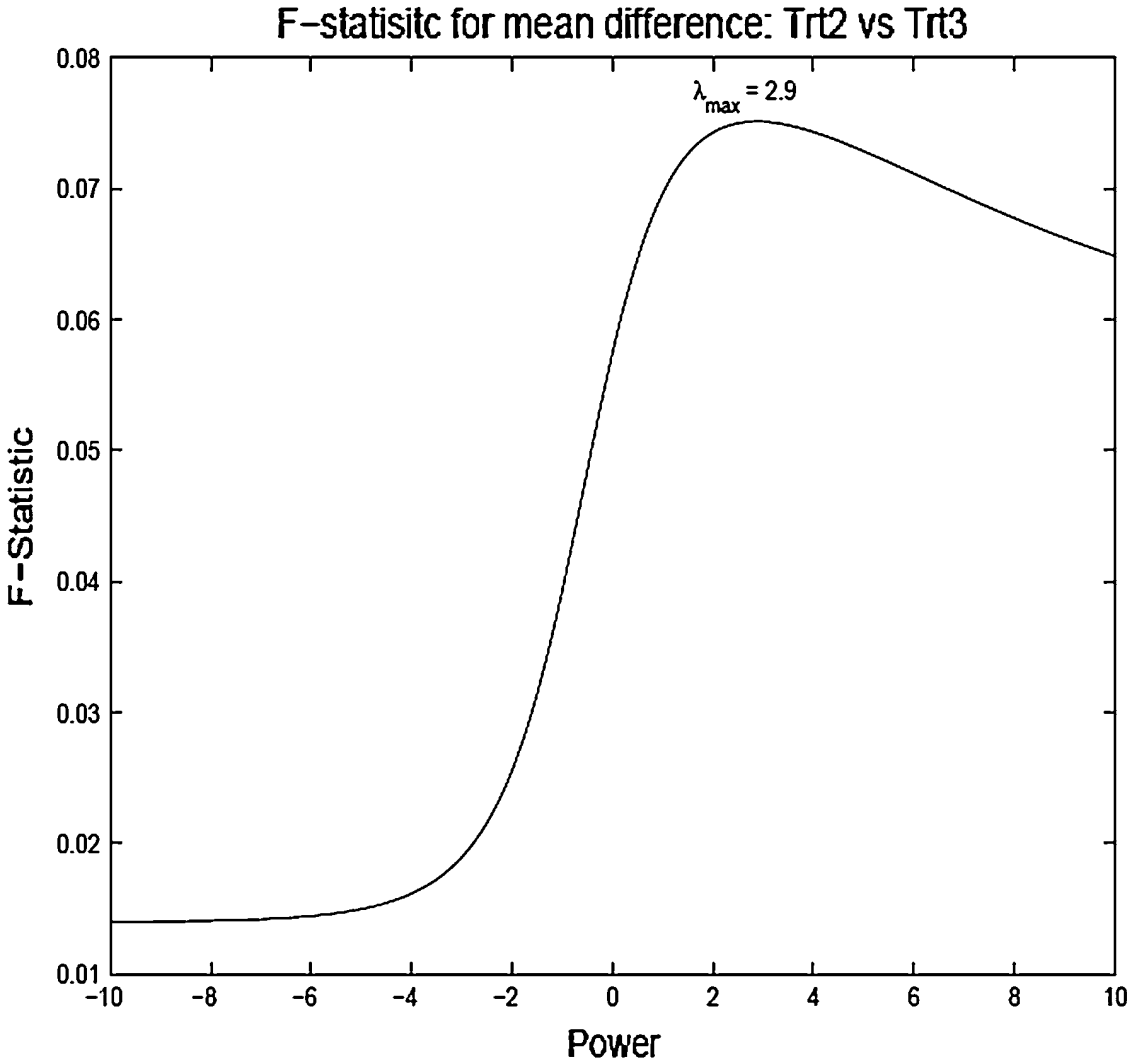
2B vs 2C

**Fig. 5:**
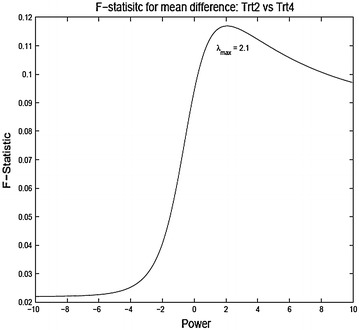
2B vs 2D ANOVA F-statistic for mean difference

Of course the model will be indexed by the exponent $\lambda$ of the power transformation. Figure 3, 4 and 5 show the plot of the F-statistic for testing equality of the treatment means as a function of $\lambda$. Generally, the unique mode $\lambda _{max}$ lies well right of $\lambda =1$ ($\lambda =$ 1.6, 2.1 and 2.9 for treatment comparisons (2A, 2C), (2B, 2C), and (2B, 2D), respectively). This reflects the general right skewed nature of the data. A power transformation bigger than one may be desirable as it may be argued that scoring system which puts more weight on higher score corresponding to more severe symptoms is indeed more apt to detect treatment differences. Although the figures show that higher power may be gained by making appropriate power transformation, none of the F-statistic values are significant for this particular example. This may be due to the naive assumption of equal variances for the treatment groups (in this particular example: Bartlett’s K-squared = 2.189, df = 3, p value = 0.5341). Given that the observations have been transformed through nonlinear transformations, the assumption of equal variances can be rather uncomfortable. The procedure can be made much more efficient by treating the variances as unknown and possibly unequal. This in this simple model is analogous to the classic Behren–Fisher problem for testing equality of means of two normal populations with unequal variances.

### Unequal variances

Even though there are no optimal procedure for dealing with the Behren–Fisher problem, a novel and appealing way is to use the recent ideas of generalized confidence intervals. The concept is due to Weerahandi (1993) and the basic idea is as follows. Let *X* be a random vector whose distribution depends on $\delta$, a scalar parameter of interest and $\eta$, a nuisance parameter. Furthermore, let *x* denote the observed value of *X*, the already obtained data on *X*. Then a *generalized pivot statistic*$g(X; x, \theta )$ is a statistic satisfying the following conditions:

1. The distribution of $g(X; x, \theta )$ is free from any unknown parameters.
2. The observed value of $g(X; x, \theta )$, i.e., $g(x; x, \theta )$, is equal to $m(\theta )$, the parameter of interest.

Confidence intervals for $m(\theta )$ are obtained using the percentiles of $g(X; x, \theta )$ and are known as *generalized confidence intervals*. The coverage of such a confidence interval conditional on the data is equal to the nominal level but the overall coverage may not be exactly equal to the nominal level. In fact, the coverage could depend on unknown parameters. However, for the Behren–Fisher problem the coverage is remarkably close to the nominal level for a variety of parameter configurations; see Weerahandi (1993), Weerahand (1995) for details. In general, the percentiles of a generalized pivot statistic will have to be numerically obtained, perhaps by simulation.

In our case, the parametric function of interest when comparing treatment B and C is

$$\begin{aligned} m(\mu _B, \mu _C, \sigma _B, \sigma _C) = |\mu _B - \mu _C|, \end{aligned}$$

where $\mu _B, \mu _C, \sigma _B, \sigma _C$ are the means and standard deviations of the respective populations. Because the ANOVA statistics perform optimally for normal distribution, we will do the mean comparison with the target that the power transformation is increasing efficiency by bringing the empirical distribution closer to normal distribution. In this context we can construct a generalized pivot for the treatment mean differences pretending we have normality for the data. The generalized pivot would be

3.5 $$\begin{aligned} T = {\bar{x}}_{A} - {\bar{x}}_{B} - \frac{({\bar{X}}_{A} - \mu _A)}{\sigma _A/\sqrt{n_A}}\frac{\sigma _A}{S_A} \frac{s_A}{n_A} + \frac{({\bar{X}}_{B} - \mu _B)}{\sigma _B/\sqrt{n_B}}\frac{\sigma _B}{S_B}\frac{s_B}{n_B}, \end{aligned}$$

where ${\bar{x}}_{A},{\bar{x}}_{B},s_A,s_B$ are the observed sample means and standard deviations and ${\bar{X}}_{A},{\bar{X}}_{B},S_A,S_B$ are the corresponding population quantities.

**Fig. 6:**
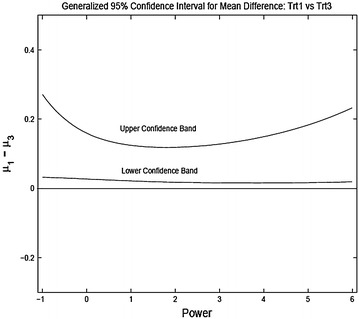
2A vs 2C

**Fig. 7:**
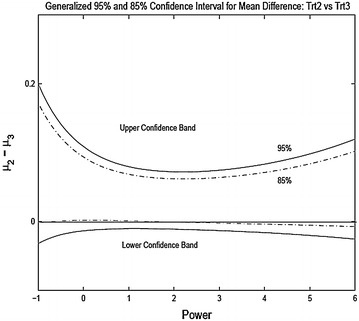
2B vs 2C

**Fig. 8:**
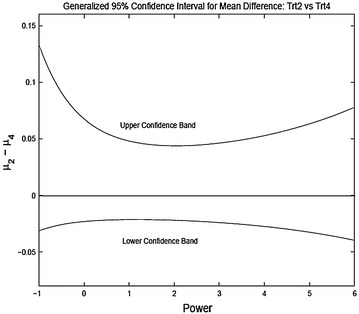
2B vs 2D generalized 95 % confidence interval

We present the results for three pairwise comparisons, 2A vs 2C, 2B vs 2C and 2B vs 2D. Figures 6, 7 and 8 shows the 95 % generalized confidence bands for the three pairwise comparisons where the confidence limits are plotted as a function of the power transformation. For comparing 2A to 2C the confidence intervals do not include zero showing significant difference at 5 % level. For comparing treatment 2B and 2C, the confidence limits do include zero. However, for 85 % confidence bands the confidence intervals do not include zero for $\lambda \in [-1, 2]$ and the shortest length of the interval in obtained for $\lambda = 2$. For comparing treatments 2B and 2D the intervals contain zero for all reasonable levels.

Comparing the results of the nonparametric tests and the parametric procedures for unequal variances one sees similarity in the treatment comparisons. However, it seems that the parametric tests with power transformation may have more power in detecting treatment differences. For example, when comparing the treatment 2B and 2C, the nonparametric test is not significant even though much more significant than the naive two sample *t*-test. But the generalized confidence procedure returns a p value much smaller than the nonparametric test.

## Conclusion

In this article we have investigated responsiveness as measures of treatment effect on ordinal scores. We have tried to understand the effect of transforming the ordinal scores through a power transformation with the objective of attaining a modified scoring system which gives the most significant results as opposed to power transformation with the goal of achieving normality. We used the quality of life data as the primary example and illustrated our methods based on that data. There are several interesting observations that can be made from the results. The test statistics as a function of the exponent are usually unimodal, though not always convex. The power range giving the most significant results need not be equal to that obtained from the Box–Cox procedure. The transformed data can pick up differences in means which otherwise are insignificant in the original data. There are several issues that need to investigated further. The change of scales due to the power transformations and its impact on the analysis need to be carefully understood. The effect on the power function of the tests need to studied. Comparisons should be drawn with nonparametric tests. The overall findings of our investigation indicate that methods of analyzing QOL data that actually use the raw scores or change or percentage change from baseline tend to be highly sensitive to the method of score assignment. Thus, care must be exercised when using any method that uses change or percentage change. A proper sensitivity analysis showing robustness of any particular method for analyzing Symptoms or QOL scores to score assignment method must precede any validation of QOL instruments using the method.
